# Supplementary material for: A Likelihood Approach for Real-Time Calibration of Stochastic Compartmental Epidemic Models
Source: PLoS Comput Biol. 2017 Jan 17;13(1):e1005257. doi: 10.1371/journal.pcbi.1005257 (PMC5240920; doi:10.1371/journal.pcbi.1005257)
Supplement: S1 File — (TAR.GZ) [file pcbi.1005257.s014.tar.gz › HSPH_Online-SI-Revision/Additions/EnKF08/n100-extreme-SEITR_noisy/n100-extreme-SEITR_noisy_EnKF2_table8.pdf]

| | | | | | | | | | | | | | | | | | | | | | | | | | | | | | | |
|---|---|---|---|---|---|---|---|---|---|---|---|---|---|---|---|---|---|---|---|---|---|---|---|---|---|---|---|---|---|---|
| 1 | newly 3 weeks spec | 50.75 | 1200.48 | {0., 682.} | 2341.77 | 1.32339 | newly 3 weeks cumul | 584.434 | 8099.51 | {501., 10575.} | 1297.51 | 0.720387 | newly 5 weeks spec | 42.6203 | 399.78 | {0., 246.} | 960.036 | 1.46056 | newly 5 weeks cumul | 687.444 | 9173.87 | {532., 10878.} | 1246.95 | 0.684272 | newly 42 weeks cumul | 3753.74 | 9738.67 | {536., 10921.} | 231.003 | 0.440652 |
| 2 | newly 3 weeks spec | 102. | 1722.98 | {163., 2317.} | 1597.9 | 0.799374 | newly 3 weeks cumul | 593. | 9067.51 | {2579., 9280.} | 1432.15 | 0.908562 | newly 5 weeks spec | 36. | 976.2 | {4., 1363.} | 2655.61 | 0.963424 | newly 5 weeks cumul | 673. | 11346.4 | {3168., 12466.} | 1588.71 | 0.927944 | newly 50 weeks cumul | 712. | 15060.6 | {3470., 51392.} | 2017.76 | 0.989869 |
| 3 | newly 3 weeks spec | 169. | 1797.42 | {19., 5452.} | 1034.72 | 0.744514 | newly 3 weeks cumul | 920. | 11709.2 | {480., 44181.} | 1195.79 | 0.644966 | newly 5 weeks spec | 52. | 710.49 | {0., 1791.} | 1372.29 | 1.1057 | newly 5 weeks cumul | 1072. | 13519. | {500., 51789.} | 1187.05 | 0.642925 | newly 52 weeks cumul | 1137. | 14525.4 | {505., 56900.} | 1204.95 | 0.644026 |
| 4 | newly 3 weeks spec | 137. | 1424.59 | {49., 3001.} | 965.307 | 0.705803 | newly 3 weeks cumul | 790. | 11789. | {2184., 11031.} | 1399.2 | 0.775542 | newly 5 weeks spec | 41. | 616.96 | {1., 1874.} | 1459.9 | 1.06375 | newly 5 weeks cumul | 909. | 13252.3 | {2527., 20183.} | 1364.49 | 0.782123 | newly 50 weeks cumul | 985. | 16198.2 | {2713., 56562.} | 1550.12 | 0.818547 |
| 5 | newly 3 weeks spec | 34. | 1689.1 | {18., 1563.} | 4884.41 | 0.909861 | newly 3 weeks cumul | 171. | 8577.59 | {217., 10180.} | 4917.78 | 1.06261 | newly 5 weeks spec | 11. | 509.74 | {2., 358.} | 4574. | 0.870852 | newly 5 weeks cumul | 195. | 10014. | {231., 11275.} | 5038.06 | 1.05187 | newly 52 weeks cumul | 201. | 10684.7 | {232., 11775.} | 5218.8 | 1.06193 |
| 6 | newly 3 weeks spec | 5. | 1153.99 | {129., 1173.} | 22979.8 | 1.99785 | newly 3 weeks cumul | 60. | 6276.88 | {1528., 5312.} | 10361.5 | 1.73137 | newly 5 weeks spec | 2. | 487.54 | {19., 964.} | 24279. | 1.87001 | newly 5 weeks cumul | 63. | 7500.99 | {1556., 8353.} | 11806.3 | 1.78832 | newly 51 weeks cumul | 64. | 8838.34 | {1595., 17551.} | 13709.9 | 1.85242 |
| 7 | newly 3 weeks spec | 469. | 894.16 | {132., 2351.} | 134.217 | 0.422747 | newly 3 weeks cumul | 2142. | 8168.84 | {873., 21037.} | 305.318 | 0.426037 | newly 5 weeks spec | 221. | 266.32 | {15., 766.} | 96.5701 | 0.624581 | newly 5 weeks cumul | 2717. | 8895.52 | {1051., 22495.} | 250.389 | 0.385167 | newly 43 weeks cumul | 3226. | 9621.36 | {1169., 22777.} | 215.374 | 0.348281 |
| 8 | newly 3 weeks spec | 225. | 2211.33 | {133., 4989.} | 899.009 | 0.713438 | newly 3 weeks cumul | 1088. | 13512.4 | {4093., 29671.} | 1145.08 | 0.829916 | newly 5 weeks spec | 80. | 964.12 | {4., 1885.} | 1148.55 | 0.963381 | newly 5 weeks cumul | 1321. | 15844.5 | {5059., 50743.} | 1102.63 | 0.830076 | newly 50 weeks cumul | 1472. | 19697.7 | {5519., 67285.} | 1240.57 | 0.867819 |
| 9 | newly 3 weeks spec | 19. | 256.7 | {0., 413.} | 1303.58 | 1.05023 | newly 3 weeks cumul | 167. | 15139.6 | {623., 45068.} | 8966.83 | 1.43392 | newly 5 weeks spec | 5. | 17.63 | {0., 33.} | 358.2 | 1.0581 | newly 5 weeks cumul | 179. | 15208.8 | {733., 45076.} | 8397.79 | 1.41401 | newly 52 weeks cumul | 188. | 15222.8 | {763., 45076.} | 7998.6 | 1.39609 |
| 10 | newly 3 weeks spec | 613. | 2815.82 | {351., 5648.} | 375.726 | 0.48478 | newly 3 weeks cumul | 2661. | 10591.8 | {1816., 16260.} | 310.684 | 0.471381 | newly 5 weeks spec | 260. | 1927.73 | {96., 7065.} | 669.058 | 0.666263 | newly 5 weeks cumul | 3324. | 14853.9 | {2193., 27206.} | 358.857 | 0.493345 | newly 48 weeks cumul | 3949. | 22373.2 | {3244., 82331.} | 476.102 | 0.542935 |
| 11 | newly 3 weeks spec | 448. | 4294.33 | {207., 15224.} | 875.819 | 0.690423 | newly 3 weeks cumul | 2137. | 13652.3 | {1891., 33366.} | 550.884 | 0.604196 | newly 5 weeks spec | 237. | 2363.87 | {137., 10073.} | 920.485 | 0.740363 | newly 5 weeks cumul | 2689. | 19462.8 | {2246., 60806.} | 635.779 | 0.628035 | newly 47 weeks cumul | 3221. | 25858. | {2780., 86826.} | 710.936 | 0.644669 |
| 12 | newly 3 weeks spec | 209. | 2122.16 | {45., 4003.} | 974.057 | 0.526055 | newly 3 weeks cumul | 1086. | 5035.47 | {1091., 6296.} | 369.086 | 0.283542 | newly 5 weeks spec | 68. | 1055.13 | {1., 2689.} | 1530.66 | 0.932041 | newly 5 weeks cumul | 1294. | 7708.03 | {1124., 17669.} | 507.407 | 0.315747 | newly 48 weeks cumul | 1442. | 8929.74 | {1133., 28495.} | 536.626 | 0.340803 |
| 13 | newly 3 weeks spec | 300. | 1978.38 | {135., 4672.} | 582.533 | 0.548309 | newly 3 weeks cumul | 1508. | 12359.2 | {4086., 31100.} | 726.191 | 0.743312 | newly 5 weeks spec | 131. | 894.99 | {1., 829.} | 668.71 | 0.857814 | newly 5 weeks cumul | 1818. | 14610.4 | {4181., 36738.} | 711.165 | 0.716443 | newly 49 weeks cumul | 2109. | 17550.1 | {4186., 67750.} | 740.28 | 0.689873 |
| 14 | newly 3 weeks spec | 681. | 2945.58 | {326., 7097.} | 363.286 | 0.428286 | newly 3 weeks cumul | 2981. | 14100.3 | {2666., 39920.} | 379.101 | 0.447355 | newly 5 weeks spec | 331. | 1133.53 | {30., 4011.} | 315.562 | 0.603498 | newly 5 weeks cumul | 3718. | 17055.8 | {2789., 58800.} | 367.365 | 0.443372 | newly 42 weeks cumul | 4474. | 20782.5 | {2933., 66769.} | 375.753 | 0.458272 |
| 15 | newly 3 weeks spec | 238. | 287.11 | {0., 586.} | 120.239 | 1.08231 | newly 3 weeks cumul | 1233. | 3973.68 | {37., 5803.} | 256.806 | 0.618736 | newly 5 weeks spec | 100. | 45.34 | {0., 96.} | 88.72 | 1.41403 | newly 5 weeks cumul | 1479. | 4127.66 | {39., 6156.} | 214.055 | 0.576773 | newly 43 weeks cumul | 1667. | 4168.98 | {40., 6222.} | 185.58 | 0.547912 |
| 16 | newly 3 weeks spec | 233. | 2520.4 | {190., 4215.} | 994.515 | 0.726414 | newly 3 weeks cumul | 1269. | 14069.6 | {1566., 17298.} | 1016.05 | 0.840451 | newly 5 weeks spec | 91. | 1118.3 | {7., 1448.} | 1171.78 | 0.772632 | newly 5 weeks cumul | 1497. | 16826.9 | {1842., 23532.} | 1032.01 | 0.837276 | newly 46 weeks cumul | 1647. | 19857.9 | {1915., 28830.} | 1113.78 | 0.850648 |
| 17 | newly 3 weeks spec | 1. | 1103.65 | {12., 919.} | 110265. | 2.06418 | newly 3 weeks cumul | 62. | 9101.09 | {196., 4289.} | 14579.2 | 1.39566 | newly 5 weeks spec | 0.1 | 368.95 | {0., 172.} | 368880. | 1.9657 | newly 5 weeks cumul | 64. | 10090.4 | {199., 5203.} | 15666.2 | 1.40835 | newly 52 weeks cumul | 64. | 10526.5 | {199., 6006.} | 16347.6 | 1.41636 |
| 18 | newly 3 weeks spec | 93. | 1403.55 | {82., 2009.} | 1419.43 | 0.800245 | newly 3 weeks cumul | 605. | 7991.69 | {426., 9450.} | 1233.15 | 0.741537 | newly 5 weeks spec | 53. | 588.65 | {9., 1014.} | 1038.36 | 0.915995 | newly 5 weeks cumul | 722. | 9471.28 | {691., 10798.} | 1218.29 | 0.743258 | newly 51 weeks cumul | 795. | 11429.5 | {1801., 21572.} | 1341.34 | 0.852666 |
| 19 | newly 3 weeks spec | 465. | 1075.7 | {0., 1747.} | 253.669 | 1.1274 | newly 3 weeks cumul | 2284. | 6695.33 | {890., 20913.} | 251.38 | 0.411653 | newly 5 weeks spec | 217. | 383.4 | {0., 337.} | 226.995 | 1.74781 | newly 5 weeks cumul | 2812. | 7748.56 | {896., 21519.} | 244.681 | 0.450373 | newly 46 weeks cumul | 3336. | 8181.26 | {896., 21521.} | 223.91 | 0.481819 |
| 20 | newly 3 weeks spec | 75. | 1106.64 | {0., 1146.} | 1464.96 | 1.49227 | newly 3 weeks cumul | 401. | 15827.5 | {3378., 68735.} | 3847. | 1.25901 | newly 5 weeks spec | 14. | 282.59 | {0., 649.} | 2025.36 | 1.7018 | newly 5 weeks cumul | 459. | 16650.7 | {3463., 68738.} | 3527.6 | 1.22531 | newly 51 weeks cumul | 486. | 17109.7 | {3481., 68738.} | 3420.51 | 1.21503 |
| 21 | newly 3 weeks spec | 38. | 1808.01 | {64., 6216.} | 4658.03 | 0.917262 | newly 3 weeks cumul | 270. | 13027.4 | {2513., 38457.} | 4724.96 | 1.29407 | newly 5 weeks spec | 12. | 395.78 | {1., 837.} | 3260.33 | 0.89112 | newly 5 weeks cumul | 303. | 14278.3 | {2545., 46991.} | 4612.31 | 1.27018 | newly 52 weeks cumul | 316. | 14826.5 | {2548., 46992.} | 4591.94 | 1.27044 |
| 22 | newly 3 weeks spec | 162. | 570.14 | {4., 850.} | 345.346 | 0.874877 | newly 3 weeks cumul | 806. | 5952.16 | {1553., 10591.} | 649.248 | 0.674675 | newly 5 weeks spec | 58. | 64.63 | {0., 206.} | 157.19 | 1.74643 | newly 5 weeks cumul | 951. | 6188.53 | {1754., 10820.} | 562.605 | 0.627583 | newly 48 weeks cumul | 1042. | 6249.18 | {1754., 10854.} | 512.131 | 0.600128 |
| 23 | newly 3 weeks spec | 22. | 4213.66 | {430., 15028.} | 19053. | 1.76896 | newly 3 weeks cumul | 142. | 17694.6 | {1559., 62570.} | 12361.8 | 1.64752 | newly 5 weeks spec | 3. | 2163.03 | {55., 9616.} | 72002.3 | 2.23416 | newly 5 weeks cumul | 156. | 22872.2 | {2221., 83523.} | 14561.8 | 1.71551 | newly 49 weeks cumul | 161. | 28416.7 | {3615., 94977.} | 17550.1 | 1.80396 |
| 24 | newly 3 weeks spec | 51. | 984.75 | {146., 1544.} | 1841.98 | 1.18507 | newly 3 weeks cumul | 312. | 7733.86 | {1754., 10724.} | 2381.58 | 1.14231 | newly 5 weeks spec | 15. | 380.08 | {4., 750.} | 2454. | 1.23231 | newly 5 weeks cumul | 360. | 8699.53 | {1968., 11078.} | 2319.48 | 1.14876 | newly 51 weeks cumul | 369. | 9646.23 | {1976., 15666.} | 2517.12 | 1.18584 |
| 25 | newly 3 weeks spec | 160. | 2963.96 | {295., 10989.} | 1753.43 | 0.879158 | newly 3 weeks cumul | 871. | 9707.9 | {1234., 31394.} | 1017.78 | 0.754691 | newly 5 weeks spec | 60. | 2104.79 | {142., 10310.} | 3408.72 | 1.0436 | newly 5 weeks cumul | 1024. | 14342. | {1670., 50435.} | 1302.88 | 0.810895 | newly 47 weeks cumul | 1086. | 21665.2 | {2069., 97469.} | 1896.22 | 0.910375 |
| 26 | newly 3 weeks spec | 96. | 258.66 | {5., 506.} | 241.854 | 0.700927 | newly 3 weeks cumul | 682. | 2836.74 | {484., 4801.} | 328.698 | 0.517179 | newly 5 weeks spec | 33. | 112.2 | {0., 201.} | 328.485 | 1.299 | newly 5 weeks cumul | 784. | 3108.07 | {550., 6014.} | 309.305 | 0.507546 | newly 45 weeks cumul | 878. | 3491.14 | {574., 6130.} | 311.424 | 0.49826 |
| 27 | newly 3 weeks spec | 572. | 1617.34 | {0., 4020.} | 267.734 | 1.45932 | newly 3 weeks cumul | 2438. | 7749.2 | {883., 13029.} | 241.76 | 0.341967 | newly 5 weeks spec | 284. | 962.06 | {0., 3841.} | 335.176 | 1.47013 | newly 5 weeks cumul | 3101. | 9949.77 | {994., 20745.} | 251.159 | 0.366118 | newly 45 weeks cumul | 3756. | 13331.6 | {1049., 39965.} | 293.386 | 0.424424 |
| 28 | newly 3 weeks spec | 8. | 1409.64 | {38., 4852.} | 17520.5 | 1.8102 | newly 3 weeks cumul | 54. | 9393.76 | {369., 23838.} | 17295.9 | 1.91769 | newly 5 weeks spec | 1. | 353.63 | {5., 994.} | 35269. | 1.97113 | newly 5 weeks cumul | 61. | 10449. | {391., 27461.} | 17029.5 | 1.90429 | newly 52 weeks cumul | 63. | 10855.5 | {393., 28139.} | 17131. | 1.90517 |
| 29 | newly 3 weeks spec | 378. | 946.93 | {0., 1054.} | 270.056 | 1.02205 | newly 3 weeks cumul | 1806. | 7635.36 | {2805., 10789.} | 326.148 | 0.41478 | newly 5 weeks spec | 145. | 376.08 | {0., 467.} | 312.869 | 1.84118 | newly 5 weeks cumul | 2196. | 8594.48 | {2843., 13733.} | 295.26 | 0.353956 | newly 49 weeks cumul | 2473. | 9446.56 | {2843., 14901.} | 286.384 | 0.31822 |
| 30 | newly 3 weeks spec | 146. | 1257.37 | {12., 4190.} | 792.568 | 0.818252 | newly 3 weeks cumul | 831. | 13024.9 | {1198., 73956.} | 1469.08 | 0.696046 | newly 5 weeks spec | 47. | 503.37 | {0., 513.} | 1020.02 | 0.991912 | newly 5 weeks cumul | 975. | 14140.5 | {1372., 74629.} | 1352.16 | 0.703362 | newly 52 weeks cumul | 1051. | 17492.9 | {1471., 74884.} | 1566.34 | 0.751146 |
| 31 | newly 3 weeks spec | 292. | 2707.65 | {63., 6477.} | 861.524 | 0.704461 | newly 3 weeks cumul | 1423. | 18204.3 | {1043., 68528.} | 1194.13 | 0.818302 | newly 5 weeks spec | 119. | 575.55 | {1., 1429.} | 468.092 | 0.996478 | newly 5 weeks cumul | 1719. | 19977.5 | {1100., 78387.} | 1078.4 | 0.793491 | newly 42 weeks cumul | 1955. | 21070.3 | {1105., 79447.} | 995.165 | 0.773039 |
| 32 | newly 3 weeks spec | 532. | 2953.81 | {229., 6569.} | 483.295 | 0.671252 | newly 3 weeks cumul | 2380. | 11372.5 | {1362., 19748.} | 395.464 | 0.510509 | newly 5 weeks spec | 254. | 1949.73 | {53., 8967.} | 705.972 | 0.745659 | newly 5 weeks cumul | 3005. | 15803.9 | {1809., 31895.} | 441.711 | 0.516476 | newly 48 weeks cumul | 3596. | 22232.2 | {2597., 82645.} | 529.21 | 0.532484 |
| 33 | newly 3 weeks spec | 65. | 91.55 | {0., 294.} | 200.569 | 2.37562 | newly 3 weeks cumul | 390. | 3238.24 | {243., 3019.} | 765.333 | 0.381212 | newly 5 weeks spec | 28. | 61.04 | {0., 61.} | 284.357 | 2.15886 | newly 5 weeks cumul | 468. | 3370.36 | {243., 4132.} | 668.577 | 0.412957 | newly 52 weeks cumul | 508. | 4002. | {243., 5359.} | 742.189 | 0.453714 |
| 34 | newly 3 weeks spec | 107. | 2104.74 | {22., 2582.} | 1893.5 | 0.872171 | newly 3 weeks cumul | 548. | 7318.47 | {665., 8956.} | 1243.85 | 0.776124 | newly 5 weeks spec | 35. | 1396.46 | {1., 2844.} | 3935.31 | 1.24655 | newly 5 weeks cumul | 648. | 10448.9 | {665., 16539.} | 1521.87 | 0.811482 | newly 48 weeks cumul | 692. | 15363.2 | {665., 40628.} | 2129.45 | 0.884568 |
| 35 | newly 3 weeks spec | 10. | 1184.08 | {114., 1210.} | 11742.4 | 1.64153 | newly 3 weeks cumul | 81. | 12644.9 | {2750., 5917.} | 15512.1 | 1.74652 | newly 5 weeks spec | 0.1 | 198.78 | {5., 438.} | 198684. | 2.74664 | newly 5 weeks cumul | 82. | 13292. | {2788., 7124.} | 16110.8 | 1.77527 | newly 52 weeks cumul | 82. | 13553.1 | {2790., 7889.} | 16429.1 | 1.79063 |
| 36 | newly 3 weeks spec | 587. | 3215.09 | {0., 4362.} | 496.128 | 1.21718 | newly 3 weeks cumul | 2638. | 17894.7 | {5042., 24841.} | 581.832 | 0.642098 | newly 5 weeks spec | 275. | 2102.68 | {0., 4180.} | 715.891 | 1.44161 | newly 5 weeks cumul | 3306. | 22609.8 | {7136., 48445.} | 586.291 | 0.651721 | newly 48 weeks cumul | 3908. | 31005.2 | {9114., 87534.} | 695.358 | 0.714587 |
| 37 | newly 3 weeks spec | 138. | 1662.61 | {54., 4383.} | 1126.93 | 0.836872 | newly 3 weeks cumul | 721. | 12593.7 | {261., 34159.} | 1666.34 | 0.921848 | newly 5 weeks spec | 53. | 630.75 | {14., 1374.} | 1120.43 | 0.783185 | newly 5 weeks cumul | 854. | 14218.3 | {300., 38638.} | 1584.55 | 0.917061 | newly 49 weeks cumul | 922. | 15969.9 | {373., 51554.} | 1650.01 | 0.934598 |
| 38 | newly 3 weeks spec | 103. | 3198.61 | {83., 8092.} | 3014.63 | 1.02643 | newly 3 weeks cumul | 598. | 16446.6 | {874., 58757.} | 2653.57 | 1.02949 | newly 5 weeks spec | 29. | 1657.63 | {9., 4401.} | 5638.45 | 1.23325 | newly 5 weeks cumul | 693. | 20380.2 | {1114., 76260.} | 2843.78 | 1.05281 | newly 46 weeks cumul | 752. | 25534. | {1590., 106768.} | 3298.55 | 1.10922 |
| 39 | newly 3 weeks spec | 188. | 2063.45 | {1., 3654.} | 1070.25 | 1.14992 | newly 3 weeks cumul | 946. | 14612.8 | {582., 37870.} | 1466.68 | 0.870867 | newly 5 weeks spec | 61. | 584.17 | {0., 880.} | 958.148 | 1.42844 | newly 5 weeks cumul | 1112. | 16231.9 | {583., 56577.} | 1383.92 | 0.85546 | newly 48 weeks cumul | 1224. | 17008.6 | {583., 56588.} | 1315.52 | 0.844442 |
| 40 | newly 3 weeks spec | 395. | 2110.28 | {322., 5386.} | 448.759 | 0.608966 | newly 3 weeks cumul | 1868. | 9049.57 | {3208., 14460.} | 387.767 | 0.513089 | newly 5 weeks spec | 218. | 1255.46 | {85., 3807.} | 499.055 | 0.643582 | newly 5 weeks cumul | 2391. | 11916.7 | {3968., 27501.} | 401.074 | 0.502892 | newly 49 weeks cumul | 2892. | 16921.8 | {4568., 70694.} | 486.67 | 0.507907 |
| 41 | newly 3 weeks spec | 12. | 1184.76 | {0., 855.} | 9845. | 1.60618 | newly 3 weeks cumul | 121. | 8726.76 | {972., 9188.} | 7112.2 | 1.34244 | newly 5 weeks spec | 2. | 387.72 | {0., 174.} | 19372. | 1.50939 | newly 5 weeks cumul | 135. | 9747.7 | {1173., 14415.} | 7120.52 | 1.33387 | newly 52 weeks cumul | 137. | 10577.1 | {1282., 16680.} | 7620.48 | 1.34952 |
| 42 | newly 3 weeks spec | 675. | 1743.43 | {59., 698.} | 260.381 | 0.506187 | newly 3 weeks cumul | 2966. | 6406.25 | {425., 3011.} | 215.896 | 0.482804 | newly 5 weeks spec | 340. | 659.94 | {34., 535.} | 192.041 | 0.55568 | newly 5 weeks cumul | 3794. | 8159.23 | {531., 3629.} | 213.553 | 0.472612 | newly 43 weeks cumul | 4690. | 9979.34 | {587., 11728.} | 201.731 | 0.459534 |
| 43 | newly 3 weeks spec | 545. | 2424.07 | {570., 3397.} | 357.165 | 0.595857 | newly 3 weeks cumul | 2315. | 11254.8 | {2858., 17293.} | 396.754 | 0.616272 | newly 5 weeks spec | 245. | 1637.12 | {54., 2679.} | 603.322 | 0.807479 | newly 5 weeks cumul | 2918. | 14814. | {5869., 24626.} | 418.046 | 0.650205 | newly 48 weeks cumul | 3370. | 24991.1 | {7286., 67558.} | 649.957 | 0.768909 |
| 44 | newly 3 weeks spec | 215. | 3217.93 | {37., 16984.} | 1453.71 | 0.663417 | newly 3 weeks cumul | 1211. | 12160.2 | {1184., 46989.} | 916.712 | 0.620821 | newly 5 weeks spec | 93. | 608.99 | {1., 2760.} | 674.742 | 1.1202 | newly 5 weeks cumul | 1475. | 14216.6 | {1203., 57303.} | 879.5 | 0.589156 | newly 48 weeks cumul | 1690. | 14744.6 | {1203., 58714.} | 790.669 | 0.559776 |
| 45 | newly 3 weeks spec | 201. | 1102.59 | {3., 1945.} | 530.224 | 0.944784 | newly 3 weeks cumul | 1070. | 6621.76 | {439., 12503.} | 542.761 | 0.631722 | newly 5 weeks spec | 90. | 631.5 | {0., 1327.} | 726.089 | 1.70788 | newly 5 weeks cumul | 1275. | 8074.31 | {440., 17256.} | 560.325 | 0.633526 | newly 50 weeks cumul | 1419. | 10250.9 | {440., 17256.} | 650.736 | 0.651232 |
| 46 | newly 3 weeks spec | 138. | 1842.87 | {1., 2317.} | 1312.73 | 1.09086 | newly 3 weeks cumul | 722. | 17876.1 | {663., 83759.} | 2381.17 | 0.83102 | newly 5 weeks spec | 50. | 410.49 | {0., 339.} | 845.94 | 1.58067 | newly 5 weeks cumul | 861. | 19212.5 | {663., 84638.} | 2141.38 | 0.803042 | newly 52 weeks cumul | 942. | 19542.3 | {663., 85521.} | 1987.07 | 0.786792 |
| 47 | newly 3 weeks spec | 224. | 3625. | {52., 11433.} | 1542.08 | 0.902969 | newly 3 weeks cumul | 1089. | 12636.2 | {234., 34530.} | 1086.49 | 0.86746 | newly 5 weeks spec | 77. | 2422.73 | {14., 11015.} | 3068.84 | 1.07034 | newly 5 weeks cumul | 1298. | 18045.9 | {297., 52083.} | 1314.98 | 0.900201 | newly 47 weeks cumul | 1446. | 26680.3 | {349., 104766.} | 1766.91 | 0.960867 |
| 48 | newly 3 weeks spec | 86. | 2125.65 | {38., 1477.} | 2389.59 | 0.929156 | newly 3 weeks cumul | 493. | 11055.2 | {3889., 9265.} | 2142.43 | 1.06414 | newly 5 weeks spec | 18. | 854.52 | {0., 777.} | 4693.78 | 1.26768 | newly 5 weeks cumul | 561. | 13265. | {4266., 10122.} | 2264.52 | 1.05612 | newly 50 weeks cumul | 581. | 15461.9 | {4391., 28956.} | 2561.26 | 1.08459 |
| 49 | newly 3 weeks spec | 117. | 320.76 | {18., 561.} | 239.915 | 0.598112 | newly 3 weeks cumul | 638. | 3752.15 | {2215., 5146.} | 488.111 | 0.68205 | newly 5 weeks spec | 40. | 74.11 | {0., 216.} | 212.075 | 1.30189 | newly 5 weeks cumul | 753. | 3978.28 | {2273., 6036.} | 428.324 | 0.627971 | newly 48 weeks cumul | 828. | 4076.45 | {2298., 6414.} | 392.325 | 0.595434 |
| 50 | newly 3 weeks spec | 261. | 2011.04 | {90., 1945.} | 694.966 | 0.66967 | newly 3 weeks cumul | 1311. | 9084.9 | {415., 15403.} | 613.941 | 0.629852 | newly 5 weeks spec | 104. | 1218.35 | {51., 1113.} | 1093.91 | 0.719445 | newly 5 weeks cumul | 1579. | 11876.8 | {535., 19199.} | 672.142 | 0.644138 | newly 49 weeks cumul | 1754. | 16501.6 | {666., 28676.} | 858.843 | 0.695716 |
